# The reliable assurance of detecting somatic mutations in cancer-related genes by next-generation sequencing: the results of external quality assessment in China

**DOI:** 10.18632/oncotarget.11306

**Published:** 2016-08-16

**Authors:** Rui Zhang, Jiansheng Ding, Yanxi Han, Lang Yi, Jiehong Xie, Xin Yang, Gaowei Fan, Guojing Wang, Mingju Hao, Dong Zhang, Kuo Zhang, Guigao Lin, Jinming Li

**Affiliations:** ^1^ National Center for Clinical Laboratories, Beijing Hospital, National Center of Gerontology, Beijing, People's Republic of China; ^2^ Graduate School, Peking Union Medical College, Chinese Academy of Medical Sciences, Beijing, People's Republic of China; ^3^ Beijing Engineering Research Center of Laboratory Medicine, Beijing Hospital, Beijing, People's Republic of China

**Keywords:** next-generation sequencing, reliable assurance, cancer-related genes, somatic mutations, external quality assessment

## Abstract

To evaluate the proficiencies of laboratories utilizing next-generation sequencing (NGS) to detect somatic mutations in cancer-related genes, an external quality assessment (EQA) was implemented by the National Center for Clinical Laboratories of China in 2015. We prepared a panel of samples that comprised eight samples made by mixing synthetic mutated DNA fragments with normal human genomic DNA and one reference sample containing only genomic DNA. We validated our sample panel, and then distributed it to laboratories across China. We received complete results from 64 laboratories. The performances of 51.6 % (33/64) respondent labs were acceptable and 26.6 % (17/64) of the labs returned perfect results. In total, 449 mistakes were reported, including 201 false-negatives (201/449, 44.8 %) and 222 false-positives (222/449, 49.4 %) and 26 slightly discordant results (26/449, 5.8 %). We believe these unsatisfactory results and varied performances are mainly due to the enrichment methods used, the diverse sequencing chemistries of the different NGS platforms, and other errors within the sequencing process. The results indicate that our sample panel is suitable for use in EQA studies, and that further laboratory training in targeted NGS testing is urgently required. To address this, we propose a targeted NGS workflow with details on quality assurance procedures according to the current guidelines.

## INTRODUCTION

Cancer, one of the leading non-communicable causes of death worldwide, is a complex heterogeneous disease involving genomic alterations. Many studies have revealed the remarkable diagnostic and/or therapeutic value of identifying genomic alterations in cancer. For example, analysis of the *BRCA1* and *BRCA2* genes has been used to predict the risk of hereditary breast and ovarian cancer [1], and identification of *BCR-ABL* fusions and *EGFR* mutations inform the use of tyrosine-kinase inhibitors in the treatment of chronic myelogenous leukemia [2] and lung cancer [3], respectively. In addition, owing to the genomic heterogeneity of cancers, patients with histologically similar tumors may harbor different mutations, while patients with histologically distinct tumors may harbor similar mutations [4]. Therefore, the identification of genomic alterations is a critical step in personalized cancer care.

Traditionally, conventional techniques like Sanger sequencing, pyrosequencing, and fluorescence *in situ* hybridization have been used to identify genomic alterations in tumors. Nevertheless, the continually increasing number of clinically relevant genomic alterations has created an urgent need for higher throughput sequencing [5]. With the advent of next-generation sequencing (NGS) technologies, this issue is being addressed. Besides reducing sample quantity requirements, NGS sequencing is time-saving and cost-effective compared to traditional techniques. Furthermore, NGS technologies can detect low frequency mutations, and mutations scattered across larger genomic regions than can be analyzed using conventional molecular methods [6]. Owing to their unprecedented advantages and excellent performance in practice, NGS technologies are beginning to replace traditional molecular genetic techniques. These include Sanger sequencing, which has been the dominant approach and the gold standard for mutation detection for the past 30 years.

In the clinical laboratory, NGS approaches are generally used as diagnostic tools to provide genetic characterizations that inform the choice of a more precise medical treatment [7, 8]. In umbrella trials, NGS techniques are valuable for identifying individual genomic profiles and clustering the patients for targeted therapies. According to the Molecular Analysis for Therapy Choice (MATCH) Program conducted by the U.S. National Cancer Institute, the choice of a therapeutic agent is based on the specific molecular findings obtained using targeted NGS analysis rather than on the type of cancer [9]. However, the implementation of NGS in clinical laboratories still poses specific challenges and external quality assessment (EQA) programs are required to evaluate the results of NGS analyses from these labs. Recently, the U.S. Centers for Disease Control and Prevention (CDC), the American College of Medical Genetics and Genomics (ACMG), the Association for Molecular Pathology (AMP), and the College of American Pathologists (CAP) have defined guidelines for effective validation of NGS methods, for monitoring the analytical process, and for reporting variants [10–13]. The Next Generation Sequencing—Standardization of Clinical Testing (Nex-StoCT) Workgroup has described strategies regarding EQA for NGS testing in clinical laboratories. This group recommended use of sample types, including DNA from well-characterized cell lines, to evaluate analytic steps, except for DNA extraction. Unless derived from a tumor, most cell lines will not contain cancer specific variants. Variants present will be in high allelic ratios. CAP has initiated the development of an EQA for a methods-based NGS proficiency-testing product. Compared with analyte-based EQAs, methods-based EQAs mainly focus on evaluating specific steps rather than the entire testing system. The European Molecular Genetics Quality Network and the UK National External Quality Assessment Scheme for molecular genetics have launched a pilot methods-based EQA for NGS in Europe, but the results have not yet been published [14]. Irrespective of the strategy adopted for an EQA, serious attention should be paid to the results of NGS analyses produced by clinical laboratories.

A number of companies and clinical laboratories have recently embraced the NGS approach as a routine diagnostic method in China, and accordingly an EQA of non-invasive prenatal testing using NGS was implemented by the National Center for Clinical Laboratories (NCCL) of China. Our EQA revealed that performance varied among the participating labs [15]. Next generation sequencing was also one of the methods adopted by the participants in another EQA study by our group, wherein their detection of *EML4-ALK* fusions was evaluated [16]. To assess the proficiency of these laboratories in detecting different types of aberrations in cancer-related genes, the NCCL launched a nationwide pilot EQA in 2015 that examined their performance in detecting somatic mutations using NGS technologies. Here, we report the results of this EQA and evaluate the abilities of various laboratories to correctly identify single nucleotide variants (SNV) and small insertions and deletions (indels).

## RESULTS

### Validation of cancer-related genes panel

All the SNVs and indels included in the DNA samples were correctly detected by Beijing GenePlus using a NextSeq CN500 sequencer. However, the detection results obtained by Thermo Fisher Scientific using an Ion PGM System included only some of the expected variants due to its reportable range of target variants. The allele frequency detection reported by each group was within the acceptable range (see Materials and Methods). The allele frequencies of *KRAS* c.34G>T (p.Gly12Cys) and *IDH2* c.419G>A (p.Arg140Gln) were between 1 % and 5 % in the samples that included them, and the allele frequencies of all other variant-alleles within samples were greater than 10 %. No false-negatives or false-positives were reported by either group. Allowing for their detectable ranges, the two laboratories successfully sequenced our panel of DNA samples and detected the included mutations in cancer-related genes using different dominant NGS platforms. The results of the validation of our panel of DNA samples are summarized in Table 1.

**Table 1:** The intended results and validation results of the EQA panel

Sample No.	Gene	Transcript ID	Variant	Exon	Allele Frequency (AF) (%)	Validated using Nextseq CN500		Validated using Ion torrent PGM	
						Variant	AF (%)	Variant	AF (%)
**1501**	EGFR	NM_005228.3	c.493C>T (p.Arg165Trp)	4	>50	NM_005228.3(EGFR): c.493C>T(p.Arg165Trp)	55.2	ND	ND
	FGFR3	NM_000142.4	c.1948A>C (p.Lys650Gln)	14	>50	NM_000142.4(FGFR3): c.1948A>C(p.Lys650Gln)	73.1	NM_000142.4(FGFR3): c.1948A>C(p.Lys650Gln)	95.6
	IDH1	NM_005896.2	c.395G>A (p.Arg132His)	4	>50	NM_005896.2(IDH1): c.395G>A(p.Arg132His)	66.1	NM_005896.2(IDH1): c.395G>A (p.Arg132His)	90.9
**1502**	FGFR2	NM_000141.4	c.1124A>G (p.Tyr375Cys)	9	>50	NM_000141.4(FGFR2): c.1124A>G(p.Tyr375Cys)	69.3	NM_000141.4(FGFR2): c.1124A>G (p.Tyr375Cys)	91.0
	PTPN11	NM_002834.3	c.182A>G (p.Asp61Gly)	3	>20	NM_002834.3(PTPN11): c.182A>G(p.Asp61Gly)	27.3	NM_002834.3(PTPN11): c.182A>G (p.Asp61Gly)	52.0
	TP53	NM_000546.5	c.448_459del12(p. Thr150_Pro153del)	5	>50	NM_000546.5(TP53):c.448_459 del12(p.Thr150_Pro153del)	60.3	ND	ND
**1503**	BRAF	NM_004333.4	c.1799T>A(p. Val600Glu)	15	>20	NM_004333.4(BRAF): c.1799T>A(p.Val600Glu)	31.8	NM_004333.4(BRAF): c.1799T>A (p.Val600Glu)	71.1
	EGFR	NM_005228.3	c.2573T>G(p. Leu858Arg)	21	>50	NM_005228.3(EGFR): c.2573T>G(p.Leu858Arg)	51.5	NM_005228.3(EGFR): c.2573T>G(p.Leu858Arg)	65.8
	FLT3	NM_004119.2	c.2520_2521insGGATCC(p.Ser840_ Asn841insGlySer)	20	>50	NM_004119.2(FLT3): c.2520_2521 insGGATCC(p.Ser840_ Asn841insGlySer)	51.7	NM_004119.2(FLT3): c.2520_2521 insGGATCC(p.Ser840_ Asn841insGlySer)	87.7
	KRAS	NM_033360.3	c.34G>C(p. Gly12Arg)	2	>10	NM_004985.3(KRAS): c.34G>C(p.Gly12Arg)	17.4	NM_033360.3(KRAS): c.34G>C (p.Gly12Arg)	49.8
**1504**	EGFR	NM_005228.3	c.2237_2254del18 (p.Glu746_Ser 752delinsAla)	19	>50	NM_005228.3(EGFR):c.2237_2254del18 (p.Glu746_Ser752delinsAla)	51.7	NM_005228.3(EGFR):c.2237_2254del18 (p.Glu746_Ser752delinsAla)	83.3
	ERBB2	NM_001005862.2	c. 2173_2174 delTTinsCC (p.Leu725Pro)	19	>50	NM_001005862.2(ERBB2): c.2173_2174delTTinsCC (p.Leu725Pro)	57.0	NM_001005862.2(ERBB2): c.2173_2174delTTinsCC (p.Leu725Pro)	82.5
	NOTCH1	NM_017617.3	c.5965G>A(p. Asp1989Asn)	32	>50	NM_017617.3(NOTCH1): c.5965G>A(p.Asp1989Asn)	65.7	ND	ND
	NRAS	NM_002524.4	c.35G>A(p. Gly12Asp)	2	>20	NM_002524.4(NRAS): c.35G>A(p.Gly12Asp)	48.7	NM_002524.4(NRAS): c.35G>A(p.Gly12Asp)	76.6
	PDGFRA	NM_006206.4	c.1664A>G(p. Tyr555Cys)	12	>50	NM_006206.4(PDGFRA): c.1664A>G(p.Tyr555Cys)	53.3	ND	ND
**1505**	AKT1	NM_001014432.1	c.49G>A(p. Glu17Lys)	2	>50	NM_001014432.1(AKT1): c.49G>A(p.Glu17Lys)	57.5	NM_005163.2(AKT1): c.49G>A (p.Glu17Lys)	91.4
	PDGFRA	NM_006206.4	c.1681_1682ins AGAGGG (p.Arg560_Val 561insGluArg)	12	>20	NM_006206.4(PDGFRA): c.1681_1682 insAGAGGG(p.Arg560_ Val561insGluArg)	27.0	ND	ND
	KRAS	NM_033360.3	c.34G>T(p. Gly12Cys)	2	>1	NM_004985.3(KRAS): c.34G>T(p.Gly12Cys)	3.0	NM_004985.3(KRAS): c.34G>T(p.Gly12Cys)	3.9
**1506**	EGFR	NM_005228.3	c.2156G>C(p. Gly719Ala)	18	>50	NM_005228.3(EGFR): c.2156G>C(p.Gly719Ala)	56.3	NM_005228.3(EGFR): c.2156G>C (p.Gly719Ala)	85.6
	JAK2	NM_004972.3	c.1821G>C(p. lys607Asn)	14	>50	NM_004972.3(JAK2): c.1821G>C(p.lys607Asn)	79.1	ND	ND
	NRAS	NM_002524.4	c.38G>A(p. Gly13Asp)	2	>50	NM_002524.4(NRAS): c.38G>A(p.Gly13Asp)	55.4	NM_002524.4(NRAS): c.38G>A (p.Gly13Asp)	87.9
	GNAS	NM_000516.5	c.601C>A(p. Arg201Ser)	8	>20	NM_000516.4(GNAS): c.601C>A(p.Arg201Ser)	46.3	NM_001077488.3(GNAS): c.604C>A (p.Arg202Ser)	74.3
**1507**	HRAS	NM_005343.2	c.181C>A(p. Gln61Lys)	3	>50	NM_005343.2(HRAS): c.181C>A(p.Gln61Lys)	81.1	NM_005343.2(HRAS): c.181C>A (p.Gln61Lys)	91.6
	JAK2	NM_004972.3	c.1821G>C(p. Lys607Asn)	14	>50	NM_004972.3(JAK2): c.1821G>C(p.Lys607Asn)	63.7	ND	ND
	KIT	NM_000222.2	c.1676_1681 delTTGTTG (p.Val559_ Val560del)	11	>20	NM_000222.2(KIT): c.1676_1681delGTTGTT (p.Val559_Val560del)	49.9	NM_000222.2(KIT): c.1676_1681delTTGTTG (p.Val559_Val560del)	83.1
	PIK3CA	NM_006218.2	c.1624G>A(p. Glu542Lys)	10	>10	NM_006218.2(PIK3CA): c.1624G>A(p.Glu542Lys)	12.1	NM_006218.2(PIK3CA): c.1624G>A (p.Glu542Lys)	26.2
**1508**	BRAF	NM_004333.4	c.1405_1407del3 (p.Gly469del)	11	>20	NM_004333.4(BRAF): c.1405_1407del3(p.Gly469del)	30.6	ND	ND
	IDH2	NM_002168.2	c.419G>A(p. Arg140Gln)	4	>1	NM_002168.2(IDH2): c.419G>A(p.Arg140Gln)	2.3	NM_002168.2(IDH2): c.419G>A(p.Arg140Gln)	3.0
	JAK2	NM_004972.3	c.1849G>T(p. Val617Phe)	14	>50	NM_004972.3(JAK2): c.1849G>T(p.Val617Phe)	66.8	NM_004972.3(JAK2): c.1849G>T (p.Val617Phe)	89.2
**15NC**	NA	NA	NA	NA	NA	NA	NA	NA	NA

ND, not detected; NA, not applicable.

### Panel distribution and response

**Figure 1:**
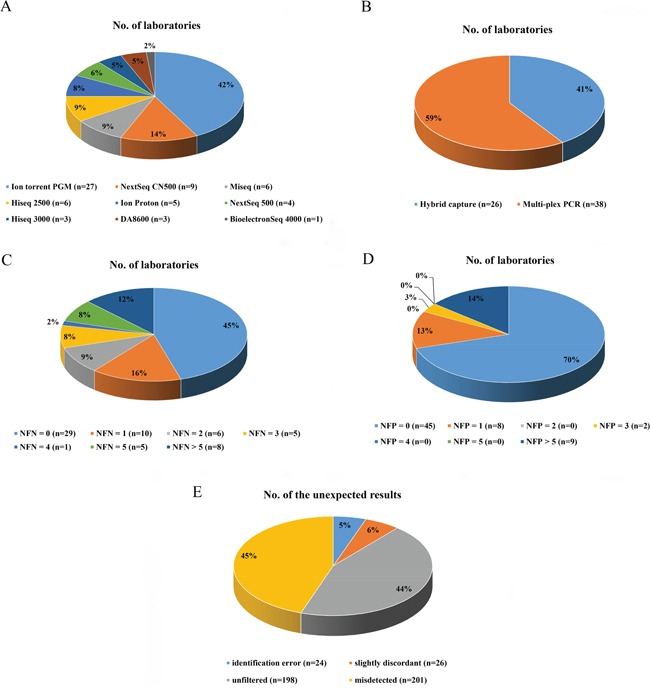
The distributions of laboratories based on differing characteristics The distributions of laboratories using specific sequencing platforms and enrichment methods are shown in **A.** and **B.**, respectively. The distributions of laboratories with different results of false-negatives and different results of false-positives are shown in **C.** and **D.**, respectively. The distribution of different types of unexpected results is shown in **E**. NFN, number of false-negatives; NFP, number of false-positives.

Seventy-five reports were received from 109 clinical laboratories before the cutoff date. Among these responses, ten laboratories did not report their detectable ranges which were necessary for analyzing our samples, and one commercial laboratory returned an incomplete dataset. Consequently, datasets from 64 laboratories, including 31 hospital or clinical laboratories and 33 commercial laboratories, were analyzed in this study. The panel was tested by participants using different next-generation sequencing approaches. The most commonly used platform was the Ion PGM System (Thermo Fisher Scientific Inc., Waltham, Massachusetts, USA) (27/64, 42.2 %), followed by the NextSeq CN500 (Hangzhou Berry Genomics, Hangzhou, China) (9/64, 14.1 %), the MiSeq (Illumina Inc., San Diego, California, USA) (6/64, 9.4 %), the HiSeq 2500 (Illumina) (6/64, 9.4 %), the Ion Proton System (Thermo Fisher Scientific Inc) (5/64, 7.8 %), the NextSeq 500 (Illumina) (4/64, 6.3 %), the HiSeq 3000 (Illumina) (3/64, 4.7 %), the DA8600 (Daan, Guangzhou, China) (3/64, 4.7 %), and the BioelectronSeq 4000 (CapitalBio, Beijing, China) (1/64, 1.6 %). Notably, 22 of 31 clinical/hospital labs (71.0 %) utilized Ion PGM/Proton instruments while 19 of 33 commercial labs (57.6 %) used Illumina platforms. Target enrichment was done using hybrid capture in 26 of the laboratories, whereas the remaining 38 laboratories employed the multiplex PCR method. All laboratories declared that the reported results had met their internal quality control standards. Figure 1 shows overviews of the various platforms and target enrichment methods used by the participating laboratories.

### NGS testing performance

**Figure 2:**
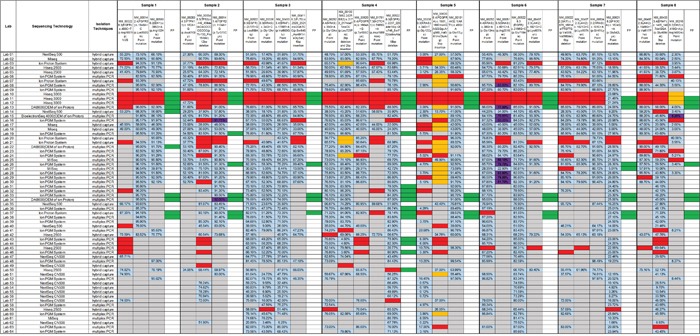
The performances of the 64 participating laboratories The distributions of results are indicated by the columns of samples between the darkest vertical lines. Within each column, the components of variants contained in the sample and the false-positives detected by the participants are shown. An open box indicates no response from the laboratory; a blue box indicates a concordant result; an orange box means a slightly discordant response; a red box indicates a false-negative result; a green box indicates a false-positive result; a purple box indicates a correct variant having different description; and a grey box indicates no response was required because a variant fell outside the specific detectable range. The allele frequencies reported are shown inside the boxes. VUS, variant of uncertain significance; FP, false-positive.

The results submitted by the participants were compared with the expected reference results, and the overall performances of the laboratories were evaluated. The results were judged to be either acceptable or improvable based on the scoring system (see Materials and Methods). Twelve results different from the expected variant descriptions in ClinVar were reported and deemed to be correct because of their availability in the dbSNP database (Figure 2).

**Figure 3:**
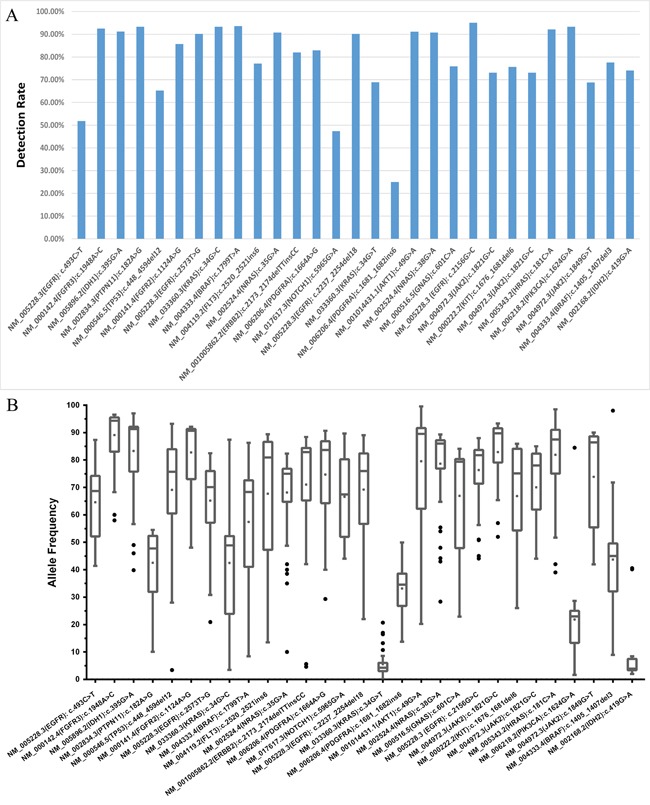
The detection rate and distribution of allele frequencies for each variant in the sample panel The detection rate of each variant identified in each sample is shown in **A.**, and **B.** describes the distribution of allele frequencies for each variant. All the variants were identified with a median frequency over 10 %, except *KRAS* c.34G>T (p.Gly12Cys) and *IDH2* c.419G>A (p.Arg140Gln) which were detected with frequencies around 3 % in samples 1505 and 1508, respectively.

The performances of 51.6% of laboratories (33/64) were found to be acceptable, and 26.6 % of these laboratories (17/64) correctly identified all the mutations within our panel of DNA samples. The results reported by the remaining 48.4% of the participants (31/64) were classified as improvable based on our criteria. Statistically, there was no significant difference between the performance of hospital/clinical labs as a group and the commercial labs as a group (*p* = 0.079). The performances of all 64 laboratories are summarized in Figure 2. The detection rates and the distributions of allele frequencies reported for each variant are shown in Figure 3.

In total, 449 mistakes were reported, including 201 false-negatives (201/449, 44.8 %) and 222 false-positives (222/449, 49.4 %) (Table 2) and 26 slightly discordant results (26/449, 5.8 %) (Table 3). It was noteworthy that 77.1 % of the false-negatives (155/201) came from only 12 laboratories and 93.7 % of the false-positives (208/222) came from only 9 participating labs, and the false-negatives or false-positives were reported in more than 5 samples by each lab involved. No false-negatives were found in the results from 45.3 % of the labs (29/64), and no false-positives were found in the results from 68.8 % of the labs (44/64). The distribution of false-negatives and false-positives reported by the participants is shown in Figure 1. Table 2 shows details regarding the false-positives. We observed that 10.8 % (24/222) of the false-positive results involving eight variants included in the designed panel were reported in the samples that did not contain them and 36.0 % (80/222) of the false-positive results involving 4 variants in NA12878 that were not included on the synthetic fragments were reported (Table 2). We also assessed the false-negative results of different combinations of target-enrichment strategies and sequencing platforms. Among labs using multiplex PCR as the enrichment method, 88 (88/159, 55.3%) false-negatives were reported by those which utilized Illumina platforms and 47 (47/587, 8.0 %) false-negatives were reported from labs adopting semiconductor sequencing systems. Among labs using hybrid capture strategies, 14 (14/383, 3.7 %) false-negatives came from laboratories using Illumina platforms and 52 (52/116, 44.8 %) false-negatives were reported by those adopting semiconductor sequencing systems.

**Table 2:** The results of false-positives

Gene	Transcript ID	Variant	No. of data sets	Sample No.	Variant in NA12878 (Y/N)	Included in EQA panel (Y/N)
FGFR2	NM_000141.2	c.1124A>G (p.Tyr375Cys)	5	1501/1505/1506	N	Y/1502
FLT3	NM_004119.2	c.2520_2521insGGATCC (p.Ser840_Asn841insGlySer)	5	1501/1502/1504/ 1506/1507	N	Y/1503
SMARCB1	NM_003073.2	c.1119-41G>A(p.?)	1	All	N	N
	NM_003073.3	c.215C>A (p.Thr72Lys)	1	1501	N	N
STK11	NM_000455	c.1062C>G (p.Phe354Leu)	1	All	N	N
	NM_000455.4	c.1086C>T (p.Tyr362Tyr)	1	1506	N	N
	NM_000455.4	c.1085A>T (p.Tyr362Phe)	1	1505	N	N
ATM	NM_000051.3	c.3912A>G (p.(=))	1	1501/1505/1506/ 1507/1508	N	N
FGFR3	NM_001163213.1	c.1936A>G (p.Asn646Asp)	1	1501/1505	N	N
	NM_000142.4	c.1953G>A (p.(=))	2	All	Y	N
	NM_001163213.1	c.1959G>A (p.Thr653Thr)	1	All	N	N
IDH1	NM_005896.3	c.353C>T (p.Pro118Leu)	1	1501/1505/ 1506/1508	N	N
	NM_005896.2	c.394_395CG>GT (p.Arg132Val)	2	1502/1504/1508	N	N
RB1	NM_000321.2	c.2009T>C (p.Leu670Pro)	1	1501/1505/1508	N	N
SMAD4	NM_005359.5	c.767A>T (p.Gln256Leu)	2	1501/1504/1505/ 1506/1507/1508	N	N
TP53	NM_000546.5	c.215C>G (p.Pro72Arg)	4	All	Y	N
	NM_000546	c.460G>T (p.Gly154Cys)	2	1505/1506	N	N
	NM_000546.5	c.455C>G (p.Pro152Arg)	1	1502	N	N
	NM_000546	c.474C>T (p.Arg158=)	3	1502	N	N
	NM_000546.5	c.453C>G (p.Pro151=)	1	1502	N	N
	NM_000546.5	c.797G>T (p.Gly266Val)	1	All	N	N
	NM_000546	c.458C>T (p.Pro153Leu)	3	1502	N	N
EGFR	NM_005228.3	c.2361G>A (p.(=))	3	All	Y	N
	NM_005228.3	c.837_838delGAinsCG (p.Asn280Asp)	1	1501/1502/1503/ 1507/1508	N	N
	NM_005228.3	c.2236_2254delGAATTAAGAGAAGCAACAT (p.Glu746fs)	2	1504	N	N
	NM_004985.3	c.2573T>G (p.Leu858Arg)	1	1504	N	Y/1503
	NM_005228	c.2156G>C (p.Gly719Ala)	1	1505	N	Y/1506
	NM_005228.3	c.2237_2254del18 (p.Glu746_Ser752delinsAla)	1	1505	N	Y/1504
ERBB2	NM_004448.3	c.3508C>G (p.Pro1170Ala)	1	All	Y	N
	NM_004448.3	c.2580A>G (p.(=))	1	1501/1504/1505/ 1506/1507/1508	N	N
	NM_004448.3	c.2263_2264delTTinsCC (p.Leu755Pro)	1	1505	N	Y/1504
	NM_004448.3	c.1558T>A (p.Cys520Ser)	1	1505	N	N
NPM1	NM_002520.5	NM_002520.6:()	1	1502/1503/ 1507/1508	-*	N
KRAS	NM_004985	c.33_34insGGAGCT (p.Ala11_Gly12insGlyAla)	3	1503/1505	N	N
	NM_033360.3	c.148A>C (p.Thr50Pro)	1	1505	N	N
BRAF	NM_004333.4	c.1799T>A (p.Val600Glu)	1	1504	N	Y/1503
GNAS	NM_080425.3	c.2530C>T (p.Arg844Cys)	1	1505	N	N
ALK	NM_004304.4	c.3551G>A (p.Gly1184Glu)	1	1505/1508	N	N
	NM_004304.4	c.3627A>G (p.Arg1209Arg)	1	1508	N	N
NOTCH1	NM_017617.3	c.4802A>T (p.His1601Leu)	1	1505	N	N
NRAS	NM_002524.4	c.35G>A (p.Gly12Asp)	2	1505/1506	N	Y/1504
	NM_002524.4	c.359T>G (p.Leu120Trp)	1	1508	N	N
	NM_002524	c.38G>C (p.Gly13Ala)	1	1505	N	N
GNAQ	NM_002072.4	c.671C>A (p.Thr224Asn)	1	1507	N	N
KIT	NM_000222.2	c.1676T>G (p.Val559Gly)	2	1507	N	N
	NM_001093772	c.1663_1668del6 (p.555_556delValVal)	1	1507	N	N
PTPN11	NM_002834.3	c.1514T>C (p.Val505Ala)	1	1507	N	N

* variant not identified.

**Table 3:** Overview of 26 slightly discordant results

Gene	Sample No.	Transcript ID	Intended Variant	Lab	Variant Reported
ERBB2	4	NM_001005862.2	c.2173_2174delTTinsCC (p.Leu725Pro)	03	c.2174T>C(p.L725S)
				20	c.2174T>C(p.L725S)
				21	c.2174T>C(p.L725S)
				24	c.2174T>C(p.Leu725Ser)
PDGFRA	5	NM_006206.4	c.1681_1682insAGAGGG (p.Arg560_Val561insGluArg)	02	c.1676_1677insGAGGGA (p.Trp559delinsTrpArgGlu)
				03	c.1676_1677insGAGGGA (p.Trp559delinsTrpArgGlu)
				06	c.1676_1677insGAGGGA (p.Trp559_Arg560insArgGlu)
				07	c.1676_1677insGAGGGA (p.W559delinsWRE)
				08	c.1676_1677insGAGGGA (p.Trp559_Arg560insArgGlu)
				13	c.1676_1677insGAGGGA (p.Trp559_Arg560insArgGlu)
				15	c.1676_1677insGAGGGA (p. Trp559_Trp560ArgGlu)
				17	c.1676_1677insGAGGGA (p.Trp559delinsTrpArgGlu)
				18	c.1676_1677insGAGGGA (p.Trp559delinsTrpArgGlu)
				21	c.1676_1677insGAGGGA (p.W559delinsWRE)
				22	c.1676_1677insGAGGGA (p.Trp559_Arg560insArgGlu)
				23	c.1676_1677insGAGGGA (p.Trp559delinsTrpArgGlu)
				26	c.1676_1677insGAGGGA (p.Trp559_Arg560insArgGlu)
				27	c.1676_1677insGAGGGA (p.W559_R560insRE)
				28	c.1676_1677insGAGGGA (p.Trp559_Arg560insArgGlu)
				29	c.1676_1677insGAGGGA (p.Trp559_Arg560insArgGlu)
				30	c.1676_1677insGAGGGA (p.Trp559_Arg560insArgGlu)
				51	c.1676_1677insGAGGGA (p.Trp559delinsTrpArgGlu)
				60	c.1676_1677insGAGGGA (p.Trp559delinsTrpArgGlu)
IDH2	8	NM_002168.2	c.419G>A(p.Arg140Gln)	10	c.419G>T(p.R140L)
				11	c.419G>T(p.R140L)
				12	c.419G>T(p.R140L)

## DISCUSSION

Sequencing large and complex DNA samples, such as those generated in transcriptome sequencing or whole-genome sequencing experiments, is expensive and time consuming. Targeted genome sequencing is a more efficient and affordable method that focuses on higher coverage or read depth over limited regions of specific genes. Targeted sequencing of cancer related genes has been the prominent approach used by clinical laboratories for routine screening of genomic variations in cancer-patient samples [17]. However, an EQA system for targeted genome sequencing by NGS has been not available until now. The ideal EQA samples should be obtained from clinical specimens that have been tested in a clinical laboratory, and should allow all phases of the testing process to be evaluated [18, 19]. However, NGS laboratories usually analyze tumor DNA and normal DNA in parallel to find somatic alteration present in the tumor. Thus, for formalin-fixed paraffin-embedded (FFPE) treated tumor tissues, normal tissues or mononuclear cells from the same patients need to be provided simultaneously as EQA control samples. Therefore, use of FFPE clinical tissue samples for large-scale EQA studies would be nearly impossible because of the limited number of tumor and normal tissue samples available from a given patient. In addition, although FFPE tissue samples are the most commonly used samples for routine diagnostics, the fixation process usually yields degraded DNA, and sequence artifacts are frequently detected due to DNA deamination [20–26]. In light of these issues, synthetic DNA samples containing specific sequences have been used for EQA studies [18], and artificially constructed DNA samples containing clinically relevant mutations have been designed for similar performance evaluation [27, 28]. In this study, we generated a panel of DNA samples by mixing genomic DNA from a HapMap cell line with synthetic DNA fragments engineered to contain previously reported cancer-related mutations. The HapMap genomic DNA, NA12878, has been developed by the National Institute for Standards and Technology (NIST) as a certified reference material [29] and a high confidence variant call set covering 78% of the genome has been characterized [30], which makes the genetic background of the samples available. Our approach was less complicated and cumbersome than the previously reported strategy of performing site-directed mutagenesis, and many different mutations can be included in one sample by using synthetic DNA fragments [27]. We directly synthesized and purified DNA fragments of 300-500 bp harboring our desired mutations. These fragments are larger than the amplicons or sheared DNA fragments generated during the NGS library preparation process, and notably the DNA sequences flanking the mutations in these fragments are identical to the genomic DNA sequences that would flank them *in vivo*. In our panel, each DNA sample contained both wild-type alleles and the corresponding disease-associated artificial allelic variants. The results of our validation process showed that the abundance of most of the artificial allelic variants in our EQA samples were within the detection limits of all the participating laboratories. Therefore, our panel of DNA samples is a valid substitute for a panel of clinically extracted human genomic DNA samples, and is suitable for evaluating genomic variation screening in labs using NGS.

Overall, only 26.6 % (17/64) of the laboratories detected the artificially mutated alleles with no mistakes in this EQA study, and 48.4 % (31/64) of the participating labs did not produce acceptable results. This shows that the application of NGS in clinical laboratories around China is still problematic and requires improvement. To facilitate this, we analyzed the unsatisfactory results to identify causal factors within the different sample preparation and sequencing processes. The problems with the sequencing results included both excessive false-positives and excessive false-negatives.

Almost half of the incorrect results were false-positives, which are known to occur for various reasons. First, since the identification of barcodes within the sequencing reads is a critical step for ensuring that subsequent characterization of the individual samples is accurate [31], errors in identifying sequence-ligated barcodes during the de-multiplexing process will cause errors in the results. In the present study, eight of the variants that were included in some of our DNA samples were detected in samples that did not contain them, suggesting possible mistakes in DNA barcode identification (Table 2). Second, in theory, the control sample containing only extracted genomic DNA should act as a baseline reference without any of the artificial allelic variants. Hence, we believe that the results involving 4 variants in NA12878 should be unfiltered results, which could be attributed to errors during the sequencing and bioinformatics procedures. These errors typically include mononucleotide stretch errors in semiconductor sequencing platforms [32], substitution errors in Illumina instruments [33], or complete omission of the filtering step.

On the other hand, among the 201 false-negatives, 60 of the 92 incorrectly reported indels (65.2 %) came from labs utilizing the multiplex PCR method to generate multiplex amplicons in the process of library preparation. As the primers designed for multiplex PCR are crucial for this enrichment method, the failure in detection could be explained by mismatches between primers and their target DNA sequences. We also found that among the labs using multiplex PCR method, the false-negative rate using Ion Torrent platforms (8.0 %) was much lower than that using Illumina platforms (55.3 %). We speculate that the laboratories using Ion Torrent platforms always adopted ‘off the shelf’ panels offered by Thermo Fisher, which have been extensively validated and the information about mutation detection performance (e.g. the reportable range) can be obtained from the manufacturers directly. In contrast, among participants using the hybrid capture method, the false-negative rate when using Ion Torrent platforms (44.8 %) was greater than that when using the Illumina platforms (3.7 %). The reason might be that hybridization-based enrichment strategies require more bioinformatics supports than PCR-based ones [34]. More commercial software and free pipelines available for the Illumina sequencing platforms might be helpful for the labs to handle the data produced using hybrid-capture enrichment. These also presented that the validation of the NGS assays might be absent in some laboratories. Therefore, we recommend that the full validation of variant detection is indispensable for laboratories when NGS tests are developed.

Furthermore, many of the unexpected results, such as the 26 slightly discordant results, should be attributed to systemic errors. These include PCR errors during the library or template preparation process [35], GC contents bias [36, 37], and potential biases within the bioinformatics pipeline such as the signal-processing and base calling limitations of the software used [36]. Errors might also appear if the sequences of junction fragments were not aligned to NCBI build 37, which was assigned as the reference sequence in this EQA. The noticeably concentrated distribution of false-negatives and false-positives implies that errors might be caused by improper operations performed within specific labs. Therefore, good standardized operating procedures (SOPs) and well-trained staff are critical, given that mistakes can occur even with the most effective instruments if procedures are performed incorrectly.

In conclusion, we designed and conducted the first nationwide EQA of NGS-based targeted sequencing by laboratories in China. We used a mixture of synthetic and genomic DNA instead of clinical specimens as samples, and validated the suitability of our samples for use in an EQA. However, there are certain limitations to our approach. First, our samples were processed differently than the typical clinical samples usually received by these labs for routine diagnostics, in that preparation of our samples did not involve a gDNA extraction process. Hence, the evaluation regarding dealing with clinical samples in laboratory was not considered in this study. However, we provided high quality DNA for this EQA, and attention should be paid to describing the sequencing process rather than the procedure for DNA isolation within these labs. Second, although each mutation was located in a central position within a synthetic DNA fragment, the limited fragment sizes might prevent labs from using Sanger sequencing to confirm their results, because their primer binding sites may lay outside the regions of the genome included in our fragments. To shed further light on the capabilities of diagnostic labs, future EQA studies should use FFPE samples that consist of untransformed cells and cells from the same lineage that have been modified using the CRISPR/Cas9 system to harbor desired mutations. Future studies could also include more low-percentage variants to better evaluate the detection of low allele frequency mutations.

**Figure 4:**
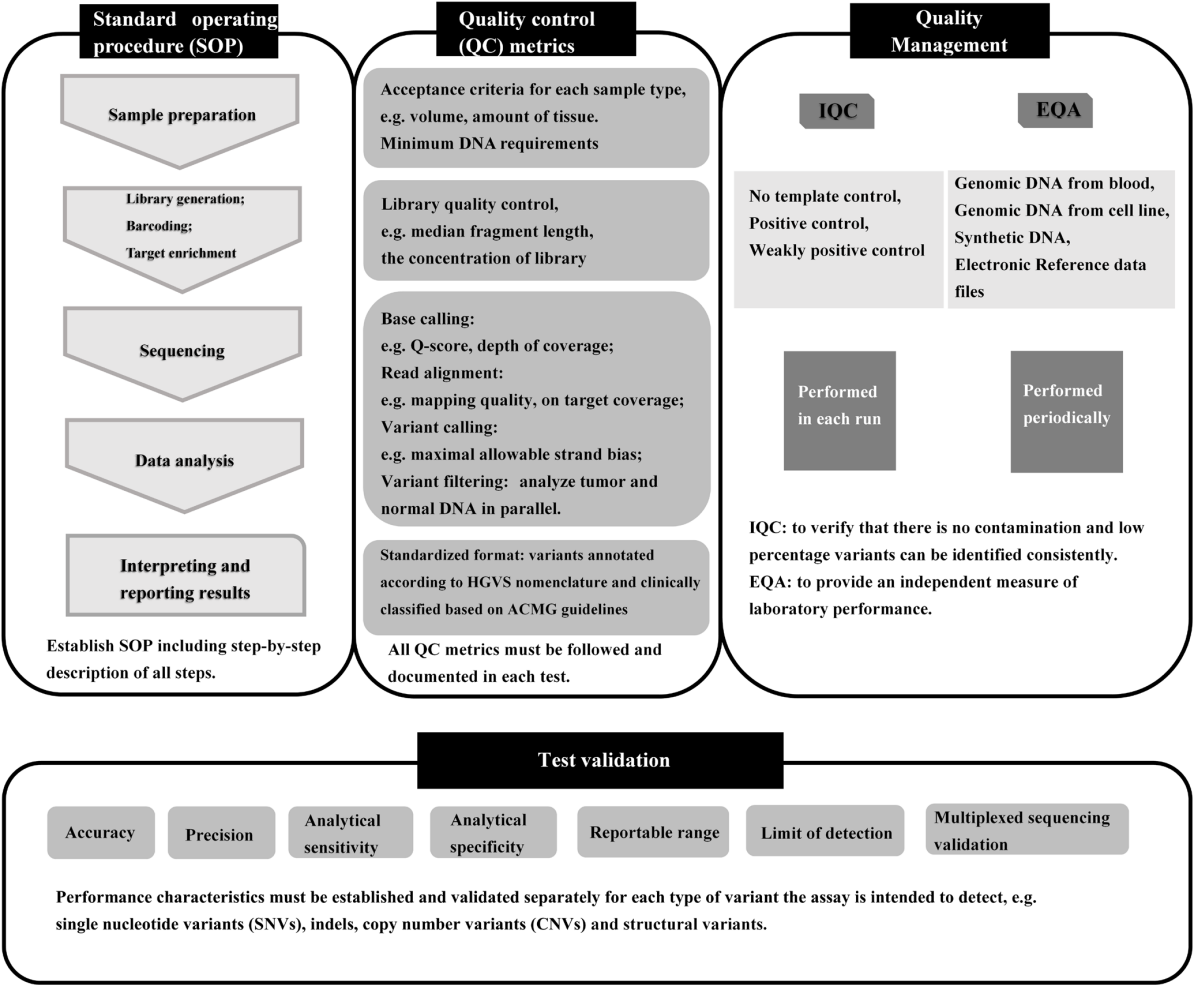
Overview of the targeted NGS workflow and quality assurance IQC, internal quality control; EQA, external quality assessment.

Our results imply an urgent requirement for improved laboratory training in the procedures of targeted NGS, likely due to the complexity of the process. Many guidelines and recommendations for standardizing NGS technologies have been produced [10–12, 38, 39], which were summarized in Figure 4. It is essential for laboratories to establish standard operating procedure (SOP), follow all quality control (QC) metrics at every step, and document the values in each test. Based on SOP and QC metrics, the NGS process should be validated to establish the expected performance characteristics within each lab. We also emphasize the importance of internal quality control (IQC) and EQA studies to verify the reliability of NGS results. As part of our EQA of labs performing targeted NGS, detailed analyses of the results were provided so that all participants became aware of the performance of various workflows and laboratories. We also provided the opportunity to retest samples for any participating labs that requested it. In the future, EQAs of targeted NGS within labs in China will be performed twice a year and the limitations of the test panel will be provided.

## MATERIALS AND METHODS

### Composition and preparation of cancer-related genes panel

The panel of eight DNA samples included mutations to 20 cancer-related genes commonly detected by clinical laboratories. The panel was prepared by the NCCL (Beijing, China) using synthetic DNA fragments and genomic DNA. Firstly, genomic DNA was extracted from a lymphoblastoid cell line (GM12878) from the International HapMap Project, which was purchased from the Coriell Cell Repositories (Coriell, New Jersey, USA). DNA from GM12878 is the same as the reference material developed by NIST, and the genome of this sample has been well characterized and is publicly available [30]. The genomic DNA was quantified using a FLUOstar Omega plate reader (BMG LABTECH, Ortenberg, Germany). Secondly, based on data from public databases ClinVar (http://www.ncbi.nlm.nih.gov/clinvar/) and the National Comprehensive Cancer Network Clinical Practice Guidelines in Oncology (NCCN Guidelines, update 2015), one SNV of uncertain clinical significance in *EGFR*, and 28 clinically significant mutations including SNVs and indels within 20 cancer-related genes were selected as candidate variants. The target gene sequences containing these variants were designed according to the curated human genome resources in the NCBI reference sequence (RefSeq) database (NCBI build 37) (http://www.ncbi.nlm.nih.gov/RefSeq/) and the sequence dataset for the HapMap sample NA12878 in the GeT-RM Browser (http://www.ncbi.nlm.nih.gov/variation/tools/get-rm/). The sizes of the desired sequences were 300-500 base pairs (bp) and the expected mutations were located in the central portions of these fragments. Synthesis of the DNA fragments was performed by Sangon Biological Technology (Shanghai, China). Recombinant plasmids containing specific mutated gene fragments were constructed and the fragments were then obtained by cleavage with restriction enzymes. Each DNA fragment was quantified using the FLUOstar Omega plate reader (BMG LABTECH, Ortenberg, Germany). Lastly, the sequences of the different synthetic DNA fragments were confirmed by Sanger sequencing and the fragments were mixed with the genomic DNA extracted from the GM12878 cell line. Specifically, 3-5 mutated fragments were pooled with the genomic DNA in controlled proportions in each sample, and the total mass of the nucleic acid was at least 1 μg. Table 1 summarizes the composition of the panel of samples: eight of the samples included synthetic DNA fragments, while one sample included only genomic DNA and acted as a control to filter out irrelevant mutations. Samples were dispensed as 30 μL aliquots into 200 μL thin-wall polypropylene PCR tubes. Each of the PCR tubes was then each placed in a 1.5 mL siliconized glass vial, in case the contents of the PCR tubes spilled during transit. The vials were labeled “NCCL NGS EQA 2015” and were randomly assigned numbers from 1-8. The samples were stored at −20°C before shipment to the laboratories.

### Validation of cancer-related genes panel

The panel of cancer-related genes was evaluated by Beijing GenePlus Technology (Beijing, China) and by Thermo Fisher Scientific Inc. (Beijing, China) using the same processes used for their routine patient sample testing.

The Beijing GenePlus group used a NextSeq CN500 sequencer (Hangzhou Berry Genomics, Hangzhou, China). DNA samples received from NCCL were first fragmented using a Bioruptor® Pico sonication system (Diagenode Inc., Denville, New Jersey, USA) and quality control was performed using an Agilent 2100 Bioanalyzer (Agilent Technologies, Palo Alto, California, USA) to ensure an average fragment size of 200-300 bp. The subsequent steps included end repair, A-tailing, and ligation with a sequencing adapter containing a unique nucleic acid barcode using a Kapa Hyper Prep Kit (Kapa Biosystems, Wilmington, Massachusetts, USA). The libraries were quantified using an ABI 7500 Real-Time PCR System (Applied Biosystems, Foster City, California, USA), and 96 libraries with different tags were pooled and quantified. The pooled library was sequenced using the NextSeq 500 High Output Kit (300 cycles) (Illumina Inc., San Diego, California, USA).

The Thermo Fisher Scientific group used an Ion PGM System (Thermo Fisher Scientific Inc., Waltham, Massachusetts, USA). Quantification of DNA samples was performed using the Qubit dsDNA BR Assay Kit and Qubit 3.0 Fluorometer (Invitrogen, Thermo Fisher Scientific Inc., Waltham, Massachusetts, USA). The processes, including multiplex PCR enrichment and library preparation, were performed using the Biometra TProfessional Standard Gradient 96 Thermocycler (Biometra, Gottingen, Germany) according to the manufacturer's instructions. The libraries were quantified using an ABI 7500 Real-Time PCR System (Applied Biosystems, Foster City, California, USA). Emulsion PCR was performed with the Ion PGM Template OT2 200 Kit using the Ion One Touch 2 system (Thermo Fisher Scientific Inc., Waltham, Massachusetts, USA). Ion sphere particles (ISP) were enriched using the E/S module and were then sequenced on the Ion PGM System using an Ion PGM™ Hi-Q™ Sequencing Kit (both from Thermo Fisher Scientific Inc., Waltham, Massachusetts, USA).

### Participating labs and data analysis

The prepared samples were shipped to 109 clinical laboratories at room temperature. All the laboratories were assigned the same coded samples and were required to perform the detection using their routine procedures. Detailed instructions for storage conditions and assay procedures were provided. The sample 15NC was specially described as normal genomic DNA extracted from normal tissues or blood cells. Laboratories were required to submit their results, including the variants and corresponding allele frequencies, within four weeks of receiving the test panel. All variants were reported following the Human Genome Variation Society (HGVS) guidelines. Since a variant might have different descriptions across different transcripts, we recommended the participants to use the reference transcripts in ClinVar database. In addition, questionnaires were sent to obtain information regarding their detectable ranges, minimum detection limits, procedures (including the platforms and reagents used for generation of DNA libraries and sequencing), databases and bioinformatics tools employed, and assay-specific quality metrics such as minimum coverage thresholds, mapping qualities, and Q scores.

To assess participant performances effectively, a set of scoring rules were established previously. Results that differed from the expected (correct) results were considered either false-negatives or false-positives. Each false-negative resulted in a deduction of 10 points from the perfect score of 100 points, whereas each false-positive resulted in loss of 5 points. A discordant result with a sequence alteration that differed within 5 bp was classified as a slightly discordant result and caused a loss of only 2 points, while a discrepancy greater than 5 bp between the reported and actual sequences resulted in losing 5 points. The variants out of the specific detectable range were not considered in the scoring process. The performance was classified as either acceptable or improvable: For labs processing a panel containing 20 or more genes, scores of 80 or more points were regarded as acceptable, and scores of less than 80 points were considered to be improvable. For labs focusing on less than 20 genes, scores of 90 or more points were necessary for an acceptable performance rating, whereas scores of less than 90 points were considered improvable. The results obtained from the laboratories were analyzed based on their detection limits and their respective reportable ranges in addition to the expected results. All statistical analysis was performed with SPSS 16.0. Performances were compared using the Fisher's exact test with a two-tailed statistical significance at *p* < 0.05.
